# Altered Intracellular Localization and Mobility of SBDS Protein upon Mutation in Shwachman-Diamond Syndrome

**DOI:** 10.1371/journal.pone.0020727

**Published:** 2011-06-13

**Authors:** Claudia Orelio, Renée M. van der Sluis, Paul Verkuijlen, Micha Nethe, Peter L. Hordijk, Timo K. van den Berg, Taco W. Kuijpers

**Affiliations:** 1 Sanquin Research and Landsteiner Laboratory of the Academic Medical Center (AMC), Department of Blood Cell Research, University of Amsterdam, Amsterdam, The Netherlands; 2 Sanquin Research and Landsteiner Laboratory of the Academic Medical Center (AMC), Department of Molecular Cell Biology, University of Amsterdam, Amsterdam, The Netherlands; 3 Emma Children's Hospital, Academic Medical Center (AMC), Amsterdam, The Netherlands; Mayo Clinic, United States of America

## Abstract

Shwachman-Diamond Syndrome (SDS) is a rare inherited disease caused by mutations in the *SBDS* gene. Hematopoietic defects, exocrine pancreas dysfunction and short stature are the most prominent clinical features. To gain understanding of the molecular properties of the ubiquitously expressed SBDS protein, we examined its intracellular localization and mobility by live cell imaging techniques. We observed that SBDS full-length protein was localized in both the nucleus and cytoplasm, whereas patient-related truncated SBDS protein isoforms localize predominantly to the nucleus. Also the nucleo-cytoplasmic trafficking of these patient-related SBDS proteins was disturbed. Further studies with a series of SBDS mutant proteins revealed that three distinct motifs determine the intracellular mobility of SBDS protein. A sumoylation motif in the C-terminal domain, that is lacking in patient SBDS proteins, was found to play a pivotal role in intracellular motility. Our structure-function analyses provide new insight into localization and motility of the SBDS protein, and show that patient-related mutant proteins are altered in their molecular properties, which may contribute to the clinical features observed in SDS patients.

## Introduction

Shwachman-Diamond Syndrome (SDS) was first described in 1964 and is a rare, hereditary disease, characterized by pancreatic insufficiency and bone marrow failure.[1], [2]. The most prominent hematopoietic defect is neutropenia, which is often accompanied by thrombocytopenia and anemia [3]–[5]. The neutropenia, together with the reported neutrophil chemotaxis defects in SDS patients, results in an increased risk of recurrent infections [5], [6]. Additionally, SDS patients have a cumulative risk of 20 to 36% of developing myeloid dysplasia (MDS) and/or acute myeloid leukemia (AML) at the age of 20 or 30 years [7].

In 2003, identification of mutations in the *SBDS* gene located at chromosome 7 in most SDS patients, provided the molecular basis for further investigations to the underlying mechanisms defective in SDS [8]. To date, several mutations have been identified but the two most common mutations are the consequence of 183–184 TA>CT and 258+2T>C genomic changes [5], [8]–[10]. These mutations are located in exon 2 and intron 2, and result in a premature stop-codon (K62X) and a frameshift mutation resulting in a stopcodon (C84fsX3) respectively [8].

Structural analysis revealed that the SBDS protein contains three domains, an N-terminal conserved FYSH domain, central helical domain and C-terminal domain with homology to an RNA-binding motif [11], [12]. The yeast ortholog of SBDS, Sdo1, was shown to interact with rRNA-processing proteins and play a role in pre-60S ribosome transport [11], [13]. In human cells SBDS was shown to interact with hsNip7, a protein required for 27S pre-rRNA cleavage and 60S subunit biogenesis [14]. Additionally, in human HeLa cells SBDS was reported to be localized to the nucleoli, a nuclear subcompartment important for ribosome processing. Altogether, these data implicate SBDS in ribosome maturation and rRNA processing. [11]–[16]. Besides a role in ribosome/RNA-related function, SBDS has been implicated in neutrophil chemotaxis and more recently SBDS has been shown to co-localize with the mitotic spindle [17], [18] indicating a potential role in chromosome segregation during mitosis. Hence, the data published so far suggest that SBDS is a multifunctional protein and proper localization and/or intracellular mobility dynamics of the SBDS protein are consequently important for fulfilling its various cellular functions.

For many proteins, including signaling proteins and transcription factors, it has been shown that intracellular localization has important consequences for their interaction partners and hence their cellular function. Aberrant localization and/or disrupted regulation have been reported to result in and/or to contribute to pathological conditions, including cancer and excessive inflammatory reactions [19]–[22]. Protein function and localization is often, rapidly and reversibly, regulated by post-translational protein modifications. Hematological abnormalities, including neutropenia and leukemia, can also be caused by gene mutations that result in altered proteins with an aberrant intracellular protein localization and function. For example, mutations in the *RPS19* gene, as observed in Diamond-Blackfan anemia patients, result in aberrant non-nucleolar localization and ribosomal defects [23]–[25]. Also, mutations in the *NPM1* gene, observed in 30% of the patients suffering from acute myeloid leukemia (AML), result in aberrant cytoplasmic protein localization that may contribute to leukemogenesis through disruption of the p14(ARF)- MDM2-p53 pathway and centrosomal duplication [26]–[28].

Similar to NPM1 and RPS19, the SBDS protein has been implicated to play an important role in ribosome function or assembly and defects in all three genes are observed in patients suffering from either neutropenia or leukemia. This raises the question whether patient-derived SBDS protein variants, similar to mutated NPM1 and RPS19 proteins, have an altered intracellular localization and possibly also different mobility properties.

To investigate this, we performed intracellular localization studies and live cell imaging with GFP- and HA-tagged SBDS proteins. Our studies reveal that the truncated patient-related SBDS proteins (SBDS-patient) preferentially localized to the nucleus and display increased nucleo-cytoplasmic trafficking as compared to full-length SBDS (SBDS-FL). Further studies allowed us to map in more detail the critical SBDS regions important for intracellular localization and trafficking and have revealed that the C-terminus of the SBDS protein is crucial for nucleo-cytoplasmic shuttling, which may be regulated by SUMOylation. Inhibition of cellular transcription or translation resulted in enhanced nucleo-cytoplasmic shuttling of the expressed SBDS proteins, supporting the presumed role of SBDS in ribosomal function. Altogether, our study provides novel genotype-function relationships and molecular insight into the function of SBDS in the Shwachman-Diamond Syndrome.

## Results

### Subcellular localization of SBDS

**Figure 1:**
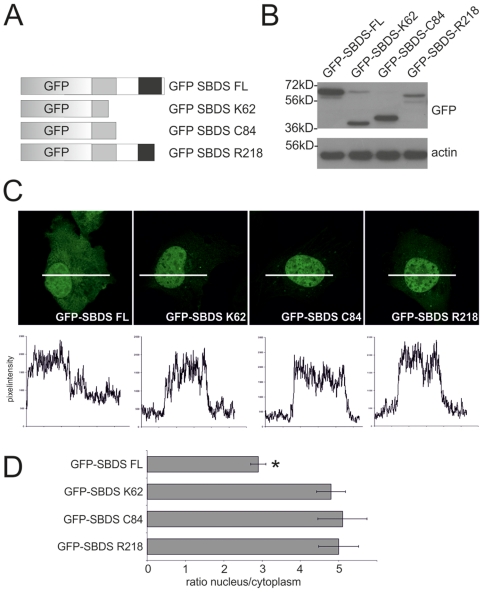
SDS-patient SBDS proteins are localized to the nucleus. (A) Schematic overview of the GFP-tagged SBDS constructs (B) Western blot analysis shows that GFP-tagged SBDS proteins have the expected molecular sizes of 59 kDa, 36 kDa, 39 kDa and 54 kDa for the GFP-SBDS-FL, GFP-SBDS-K62, GFP-SBDS-C84 and GFP-SBDS-R218 respectively. (C) Representative pictures of the intracellular localization of the GFP-tagged SBDS proteins. Bottom panel shows the GFP fluorescence intensity plots measured as indicated in the corresponding cells in the top panel. (D) Average ratio of the nuclear/cytoplasmic GFP fluorescence intensity for the different GFP-tagged constructs Asterisk indicates that the localization of the GFP-SBDS-FL is statistically significant different (p<0.001) from the SDS-patient GFP-SBDS proteins. Error bar indicates s.e.m.(FL n = 42 cells, K62 n = 52 cells, C84 n = 37 cells, R218 n = 33 cells analysed in 3–5 independent experiments).

To gain more insight into the cellular and molecular function of SBDS, we examined the subcellular localization of the full length GFP- and HA-tagged SBDS-FL protein. Also, we introduced translational stopcodons at K62, at C84 or at R218 to mimic SDS patient SBDS truncated proteins (Fig. 1A). Western blot analysis of transiently transfected HeLa cells with these GFP-tagged or HA-tagged SBDS constructs showed that these proteins are expressed and have the expected molecular sizes (Fig. 1B; Suppl. Fig. S1).

Immunofluorescence studies revealed that GFP-SBDS-FL localized both to the nucleus and the cytoplasm, with higher SBDS protein levels in the nucleus (Fig. 1C). Quantification of the protein levels in these cellular sub-compartments revealed that SBDS-FL protein levels are 3-fold higher in the nucleus as compared to the cytoplasm (Fig. 1D). In contrast, examination of the two most common patient-related SBDS mutations, (SBDS-K62 and SBDS-C84) showed that these proteins predominantly localized to the nuclear compartment with very low cytoplasmic staining (Fig. 1C). This was supported by quantification of the nuclear and cytoplasmic protein expression levels for these SBDS patient-related proteins, showing that the nuclear SBDS protein levels were 5-fold higher compared to the cytoplasm (Fig. 1D). For HA-tagged SBDS proteins and endogenous SBDS similar results were obtained (Suppl. Fig. S1, and data not shown), indicating that cellular distribution was not significantly affected by fusion to the GFP. Interestingly, the SDS-related SBDS-R218 mutant which lacks only a small part of the C-terminus [10], localized in a similar manner as the severely truncated SBDS-K62 and SBDS-C84 proteins. These data show that GFP-SBDS-FL has a significantly different subcellular distribution as compared to these patient-related SBDS protein isoforms, indicating functional differences between these SBDS proteins. Moreover, these data suggest that the SBDS C-terminus plays an important role in cytoplasmic localization.

### Patient-related SBDS proteins show enhanced nuclear import and export

To examine whether changes in intracellular trafficking contributed to the aberrant localization of the truncated GFP-tagged SBDS patient-related proteins, we performed live cell imaging experiments, including Fluorescence Recovery After Photobleaching (FRAP) and Fluorescence Loss in Intensity after Photobleaching (FLIP) experiments.

**Figure 2:**
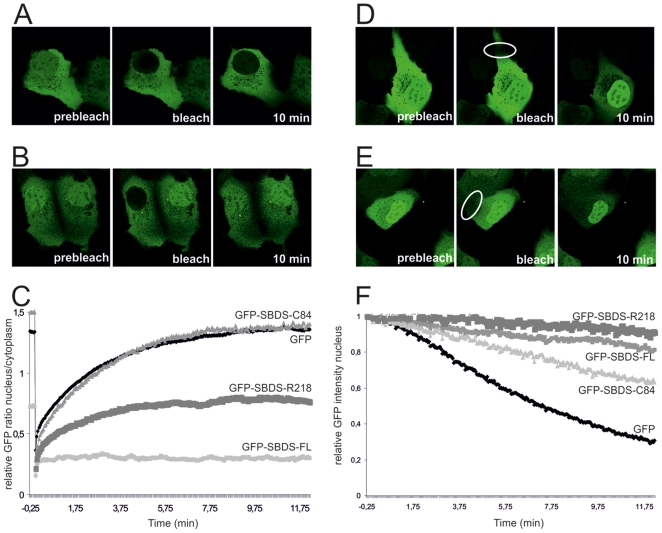
SDS-patient SBDS proteins have different intracellular mobility characteristics compared to GFP-SBDS-FL. (A) Representative GFP-SBDS-FL and (B) GFP-SBDS-C84-expressing cell for FRAP analysis prior to bleaching, at the moment of nuclear bleaching and 10 min post-bleach. (C) FRAP analysis showing the average nuclear/cytoplasmic GFP ratio for free GFP (black curve), GFP-SBDS-FL, GFP-SBDS-C84 and GFP-SBDS-R218 (grey curves); 5–9 cells per construct in 2–3 independent experiments were analysed for 10 min recovery and 12–14 cells for 5 min recovery in 3 independent experiments (not shown). (D) Representative GFP-SBDS-FL and (E) Representative GFP-SBDS-C84 expressing cell for FLIP analysis prior to bleaching, at the moment of nuclear bleaching and 10 min post-bleach (F) FLIP analysis showing the average nuclear GFP intensity for free GFP (black curve), GFP-SBDS-FL, GFP-SBDS-C84 and GFP-SBDS-R218; 7–11 cells per construct in 2–3 independent experiments were analysed.

First, FRAP analysis of GFP-SBDS-FL within the nucleus and the cytoplasm revealed that GFP-SBDS-FL protein levels were minimally recovered to the nucleus within 10 minutes post-bleaching. GFP-SBDS-FL distribution remained similar to the moment immediately after bleaching (3% of original intensity; Fig. 2A/C and Suppl. Fig. S2A). In contrast to GFP-SBDS-FL, we observed that free GFP was rapidly redistributed from the cytoplasm to the nucleus after nuclear bleaching. Within 5–6 min GFP was distributed intracellular in a similar fashion as pre-bleaching (Fig. 2C), which is consistent with the rate of free GFP mobility previously reported by others [29]. To further investigate the striking lack of nuclear import of the GFP-SBDS-FL protein, we examined the possibility that GFP-SBDS-FL nuclear import and export was hampered due to immobility in either the cytoplasm or the nucleus as the results of protein-protein interactions. Therefore, we bleached part of the cytoplasm and monitored fluorescence recovery. Within 2 minutes post-bleach full cytoplasmic recovery at the bleach area was observed, showing that GFP-SBDS-FL is mobile within the cytoplasm (Suppl. Fig. S2B), although the recovery kinetics of GFP-SBDS-FL is slower than GFP (data not shown). Similar experiments were performed to examine SBDS nuclear mobility. We observed that also within the nucleus GFP-SBDS-FL was mobile (data not shown). To examine whether eventually GFP-SBDS-FL fluorescence could be recovered after nuclear bleaching, we performed imaging for several hours after bleaching. We observed that GFP-SBDS-FL fluorescence in the nucleus recovers only after 1–2 hours post nuclear bleaching, indicating that GFP-SBDS-FL nucleo-cytoplasmic transport under steady state conditions takes place at an extremely slow rate (n = 17 cells; 2 independent experiments; data not shown).

Next, we examined the mobility of the fluorescent SBDS patient-related proteins. GFP-SBDS-K62 and GFP-SBDS-C84 showed rapid and maximal nuclear recovery (61–70% of the original nuclear intensity) within 10 minutes after photobleaching (Fig. 2B/C, and data not shown). Similar to GFP-SBDS-FL, the patient-related GFP-SBDS-K62 and GFP-SBDS-C84 proteins were mobile within the cytoplasm and nucleus (data not shown). Interestingly, the GFP-SBDS-R218 protein recovered at a slower rate than GFP-SBDS-C84 and at later time points recovery was severely hampered. This resulted in an only partial nuclear recovery of the fluorescence for GFP-SBDS-R218. In accordance with this, we observed that GFP-SBDS-R218 was partially immobile in the cytoplasm (data not shown).

To determine whether GFP-SBDS-FL and the patient-related SBDS proteins could be transported from the nucleus to the cytoplasm, we performed FLIP experiments. Interestingly, over a period of 10 minutes GFP-SBDS-FL fluorescence in the nucleus decreased to 80% of the original nuclear fluorescence intensity (Fig. 2D/F), indicating that GFP-SBDS-FL is able to cross the nuclear membrane. These results show that nuclear import and nuclear export do necessarily not take place with similar kinetics and/or similar conditions. Similar to GFP-SBDS-FL, although with slightly increased kinetics, GFP-SBDS-C84 (Fig. 2E/F) and GFP-SBDS-K62 (not shown) fluorescence in the nucleus was also clearly decreased upon repeated cytoplasmic bleaching. After 10 minutes the GFP-SBDS-C84 and GFP-SBDS-K62 fluorescence intensity was decreased to approximately 60% of the original fluorescence intensity (Fig. 2E/F). In contrast to the GFP-SBDS (truncated) proteins, free GFP fluorescence in the nucleus was rapidly lost to 30% of its original intensity upon repeated bleaching of the cytoplasm over a period of 10 minutes (Fig. 2F).

**Figure 3:**
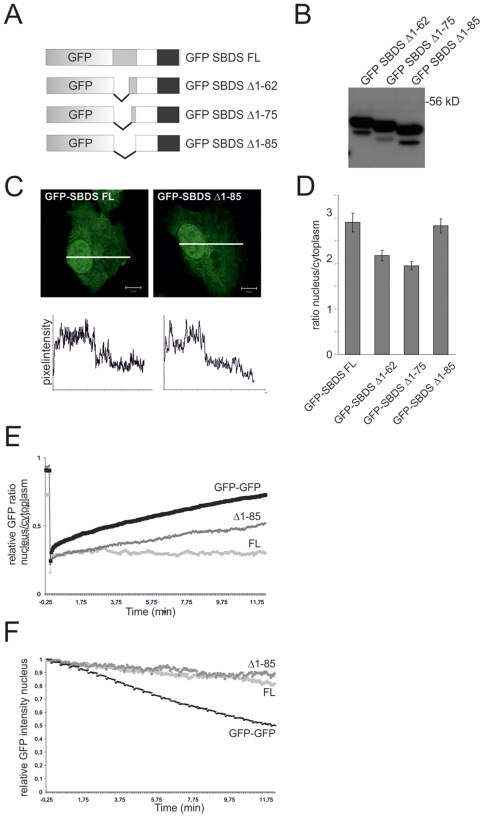
N-terminally truncated GFP-SBDS proteins localize to the cytoplasm and nucleus, but do have altered intracellular trafficking properties than GFP-SBDS-FL. (A) Schematic overview of the GFP-tagged SBDS constructs. (B) Western blot analysis shows that GFP-tagged SBDS proteins have the expected molecular sizes of 51 kDa, 49 kDa and 48 kDa for the GFP-SBDS Δ1–62, GFP-SBDS Δ1–75 and GFP-SBDS Δ1–85 respectively. (C) Representative picture of the GFP-SBDS Δ1–85 protein. Bottom panel show the GFP fluorescence intensity plots measured as indicated in the corresponding cells in the top panel. White bar represents 10 µm. (D) Average ratio of the nuclear/cytoplasmic GFP fluorescence intensity for the different GFP-tagged constructs. GFP-SBDSΔ1–62 and Δ1–75 are significantly more localized to the cytoplasm as compared to GFP-SBDS Δ1–85 (p<0.001). Error bar indicates s.e.m. (FL n = 42 cells, Δ1–62 n = 32 cells, Δ1–75 n = 28 cells, Δ1–85 n = 42 cells in 3–4 independent experiments). (E) FRAP analysis for nuclear import showing the average nuclear/cytoplasmic GFP ratio for GFP-GFP, GFP-SBDS-FL and GFP-SBDS Δ1–85. In total 6 cells per construct in 2 independent experiments were analysed. (F) FLIP analysis for nuclear export showing the average nuclear GFP intensity for GFP-GFP, GFP-SBDS-FL and GFP-SBDSΔ1–85. We analysed 7–11 cells per construct in 3 independent experiments.

To exclude the possibility that nuclear import and export of GFP-SBDS-FL was hampered due to molecular size constraints, we generated a GFP-GFP fusion protein which has a comparable molecular weight as GFP-SBDS-FL. As shown in Figure 3E/F, the GFP-GFP fusion protein was rapidly imported into and exported from the nucleus, although import/export kinetics were slower than the single free GFP (42% recovery in 10 minutes for nuclear import of GFP-GFP). Furthermore, to exclude the possibility that either SBDS protein expression levels or the position of the GFP-tag disturbs localization and mobility to great extent, we examined individual cells with different GFP-SBDS protein expression levels. We observed that expression levels did not affect GFP-SBDS protein isoform cellular behavior.

Additionally, we generated some C-terminal GFP-tagged SBDS proteins which showed similar mobility behavior as the N-terminal GFP-tagged SBDS proteins used in this study (data not shown). Altogether, this indicates that molecular size, expression level or the location of the GFP-tag does not seem to be the essential factor in preventing GFP-SBDS-FL nuclear import.

Hence, our data show that GFP-SBDS-FL is minimally imported into the nucleus under steady-state conditions, whereas only low levels of GFP-SBDS-FL are exported from the nucleus to the cytoplasm. In contrast, patient-related SBDS mutant proteins are rapidly redistributed from the cytoplasm to the nucleus and vice versa.

### SBDS protein analysis reveals critical motifs for cellular localization and trafficking

To further investigate the SBDS protein motifs and/or domains that are involved in localization and mobility, we generated several additional GFP-SBDS mutant proteins. First, GFP-SBDS N-terminally truncated constructs lacking the first 62, 75 or 85 amino acids were generated. These GFP-tagged artificial mutants are complementary to the SDS patient-derived GFP-SBDS-K62 and GFP-SBDS-C84 protein variants (Fig. 3A). Western blot analysis showed that these proteins were expressed with the correct molecular size (Fig. 3B). In contrast to the patient-related SBDS proteins, these N-terminally truncated SBDS proteins were all localized in both the nucleus and cytoplasm, in a similar manner as GFP-SDBS-FL (Fig. 3C/D). Noteworthy is that the GFP-SBDS Δ1–62 and GFP-SBDS Δ1–75 nuclear-cytoplasmic protein ratio of 2 was significantly lower as compared to the GFP-SBDS-FL and GFP-SBDS Δ1–85 nuclear-cytoplasmic ratio of approximately 3 (Fig. 3D).

These N-terminally truncated SBDS proteins showed clear differences with the full-length SBDS protein with regard to their intracellular trafficking behavior. GFP-SBDS Δ1–62, GFP-SBDS Δ1–75 and GFP-SBDS Δ1–85 were more rapidly imported into the nucleus than the GFP-SBDS-FL protein (Fig. 3E; data not shown). Nonetheless, the import rate was slower than that for GFP-SBDS-K62 or GFP-SBDS-C84 proteins (Table 1). Subsequent FLIP experiments showed that these N-terminally truncated SBDS proteins were exported from the nucleus to the cytoplasm at almost similarly slow (GFP-SBDS Δ1–62, GFP-SBDS Δ1–75) or even slower (GFP-SBDS Δ1–85) rates, when compared to GFP-SBDS-FL. The increase in export rate together with the significantly lowered nuclear-cytoplasmic ratio of the GFP-SBDS Δ1–62 and Δ1–75 as compared to GFP-SBDS Δ1–85, suggest that the protein region containing amino acids 75–85 is involved in nuclear export, although *in silico* analysis did not reveal a consensus nuclear export signal (NES) in this sequence.

**Table 1:** Overview of GFP-SBDS intracellular mobility.

Construct	nuclear import	nuclear export
GFP	**+++**	**+++**
GFP-GFP	**++**	**+++**
		
GFP-SBDS-FL	**−**	**±**
		
**SDS-patient mutations**		
GFP-SBDS-K62	**+++**	**++**
GFP-SBDS-C84	**+++**	**++**
GFP-SBDS-R218	± (*)	± (*)
		
**C-terminal truncations and mutations**		
GFP-SBDS-L234	**++**	**+**
GFP-SBDS-K225-226R	**+**	**+**
GFP-SBDS-E227Q		
		
**N-terminal truncations**		
GFP-SBDS Δ1–62	**+**	**+**
GFP-SBDS Δ1–75	**+**	**+**
GFP-SBDS Δ1–85	**+**	**±**
		
**Inhibitors**		
GFP-SBDS-FL+ actinomycinD	**±**	**++**
GFP-SBDS-FL+ cyclohexamide	**±**	**++**

Fluorescence recovery within 10 minutes: >50% = +++; 35–50% = ++; 20–35% = +; 20–5% = ±; <5% = −.

Fluorescence loss within 10 minutes: >50% = +++; 35–50% = ++; 20–35% = +; <20% = ±.

(*) GFP-SBDS-R218 is immobile in the cytoplasm and therefore nuclear import and export could not be determined properly.

Thus, these data show that the absence of the N-terminal part of SBDS does not affect cytoplasmic SBDS localization to a great extent, but strongly affects nuclear import under normal steady-state conditions.

### SBDS C-terminus dictates cytoplasmic localization

Our data indicated that the C-terminus of SBDS is required for cytoplasmic localization and/or retention, since the full-length and N-terminally truncated SBDS proteins were localized to the nucleus and the cytoplasm, whereas the C-terminally truncated patient-related SBDS protein isoforms predominantly localized to the nucleus. In line with this, GFP-SBDS-R218, which lacks only 32 amino acids of the SBDS C-terminus, also showed a prominent nuclear localization, indicating that the short stretch of amino acids 218–250, contains a pivotal localization signal.

**Figure 4:**
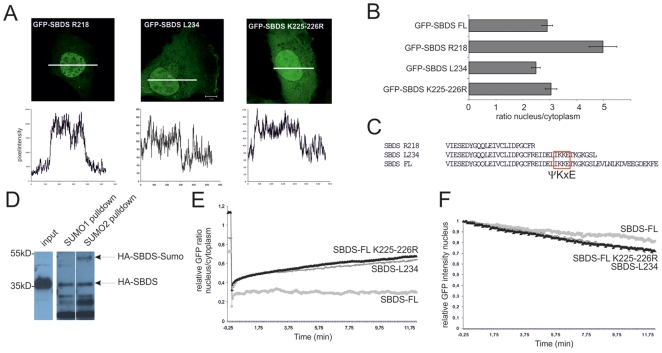
SBDS C-terminus affects intracellular transport. (A) Representative picture of the GFP-SBDS R218, GFP-SBDS-L234 and GFP-SBDS K225-226R proteins. Bottom panel shows the GFP fluorescence intensity plots measured as indicated in the corresponding cells in the top panel. White bar represents 10 µm. (B) Average ratio of the nuclear/cytoplasmic GFP fluorescence intensity for the different GFP-tagged constructs. Error bar indicates s.e.m. (FL n = 42 cells, R218 n = 33, L234 n = 37, K225-226R n = 30 cells in 3 independent experiments). (C) Alignment of the C-terminal amino acid sequence of SBDS-FL, SBDS-R218 and SBDS-L234 revealed a consensus SUMO (ΨKxE) sequence. (D) Representative Western blot analysis shows that HA-SBDS-FL can be easily detected in the input lysate and the pull-down samples. In the SUMO-2 pull-down, but not in the SUMO-1 pull-down, a 17 kDa higher molecular isoform of HA-SBDS-FL is present showing that SBDS-FL is modified by SUMO-2 (n = 3). (E) FRAP analysis showing the average nuclear/cytoplasmic GFP ratio for GFP-SBDS-L234 and GFP-SBDS-K225-226R. We analysed 6–9 cells per construct in 3 independent experiments. (F) FLIP analysis showing the average nuclear GFP intensity for GFP-SBDS-FL, GFP-SBDS-L234 and GFP-SBDS-K225-226R. 7–11 cells per construct in 2–3 independent experiments were analysed.

To explore this further, we generated the GFP-SBDS-L234 mutant, an SBDS protein lacking the last 16 C-terminal amino acids and examined its localization and mobility properties. We observed that GFP-SBDS-L234 and GFP-SBDS-FL have a similar subcellular distribution (Fig. 4A/B), indicating that the region between R218-L234 contains the essential nuclear localization motif that results in the different distribution of GFP-SBDS-R218 as compared to GFP-SBDS-FL and GFP-SBDS-L234. However, in contrast to the full-length protein, GFP-SBDS-L234 could be transported in and out of the nucleus more rapidly, suggesting that localization and mobility properties are not necessarily linked (Fig. 4E/F).

To further explore the SBDS C-terminal localization and mobility properties, we analysed the SBDS protein sequence *in silico* for potential post-translational modification consensus sequences. We observed several potential consensus sequences (ΨKxE) for Small Ubiquitin-related Modifier (SUMO) protein modification in the SBDS protein, including a SUMO consensus sequence in the C-terminus at amino acid positions 225–227 (Fig 4C). Several studies have reported that sumoylation can affect subcellular localization and protein function [30]–[35]. To explore whether SBDS can be modified by SUMOylation, we transiently expressed his-tagged SUMO-1 or his-tagged SUMO-2 together with HA-SBDS-FL in HeLa cells and performed his-SUMO pull-down assays. As shown in Figure 4D, Western blot analysis revealed that HA-SBDS-FL can be sumoylated by SUMO-2, as detected at an apparent molecular weight of 50 kDa on SDS-PAGE, consistent with an increase of 17 kDa of SBDS-FL due to the covalent binding of SUMO-2. As this protein band remains absent for a pull-down with SUMO-1, SBDS seems to be particularly sumoylated by SUMO-2. The 50 kDa sumoylated SBDS protein band could not be detected in the protein input, which suggests that only a small portion of the SBDS protein pool is sumoylated, consistent with reports on other sumoylated proteins [30]–[35]. These data thus reveal that SBDS can be sumoylated by SUMO-2, supporting the idea that post-translational modification of SBDS plays a role in subcellular mobility.

Next, we generated two GFP-SBDS protein mutants in which the C-terminal SUMO consensus site was disrupted. In the GFP-SBDS-K225-226R mutant, the two adjacent lysine residues are changed to arginine residues within the ΨKxE consensus sequence, rendering this site unsuitable for SUMO attachment at the lysine residues. Additionally, we generated a GFP-SBDS-E227Q mutant in which the potential SUMO consensus site in general was disrupted. Localization of the GFP-SBDS-K225-226R protein was observed in the cytoplasm and nucleus, similar as the GFP-SBDS-FL and GFP-SBDS-L234 proteins (Fig. 4A/B).

In the live cell-imaging experiments the GFP-SBDS-K225-226R mutant behaved identical to GFP-SBDS-L234 protein, suggesting that these two lysine amino acids are important for SBDS intracellular mobility. To confirm the importance of this site, we examined the intracellular localization and mobility of GFP-SBDS-E227Q. These data showed that GFP-SBDS-E227Q has similar intracellular localization and trafficking properties as GFP-SBDS- K225-226R (Suppl. Fig. S3), further supporting the idea that sumoylation plays a role in SBDS intracellular trafficking.

Altogether these experiments show that SBDS can be post-translationally modified by sumoylation, and that disruption of the SUMO consensus site in the SBDS C-terminus has no effect on intracellular localization, but does alter SBDS intracellular mobility.

### Physiological relevance of SBDS mobility and localization

Our data demonstrate that various SBDS protein regions contribute to its localization and/or intracellular transport, but the regulation involved in its motility within the cell remains unclear. When both the N- and C-terminus are present, the nuclear import and export of GFP-SBDS-FL are minimal under steady-state conditions. The C-terminus, including a consensus SUMO site, is important for regulating intracellular transport. Since SBDS has been proposed by several studies to exhibit RNA-binding capacity [10]–[15], [36]–[38], we examined whether RNA-related cellular processes such as transcription or translation could affect the transport of full-length SBDS protein.

**Figure 5:**
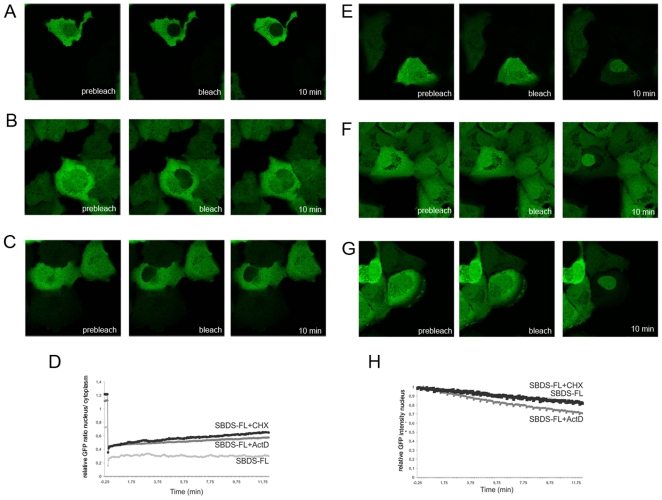
GFP-SBDS-FL intracellular transport is affected by ribosome and RNA processing inhibitors. (A) Representative GFP-SBDS-FL and (B) GFP-SBDS-FL-expressing cell in the presence of cyclohexamide (C) GFP-SBDS-FL expressing cell in the presence of actinomycinD for FRAP analysis prior to bleaching, at the moment of nuclear bleaching and 10 min post-bleach. (D) FRAP analysis showing the average relative nuclear/cytoplasmic GFP ratio for GFP-SBDS-FL (light grey curve), or GFP-SBDS-FL in the presence of actinomycinD (dark grey curve) or cyclohexamide (black curve). We analysed 8-9 cells in 2 independent experiments. (E) Representative GFP-SBDS-FL and (F) representative GFP-SBDS-FL expressing cell in the presence of cyclohexamide and (G) representative GFP-SBDS-FL expressing cell in the presence of actinomycinD for FLIP analysis prior to bleaching, at the moment of nuclear bleaching and 10 min post-bleach (H) FLIP analysis showing the average nuclear GFP intensity for GFP-SBDS-FL (lightgrey curve), GFP-SBDS-FL in the presence of cyclohexamide (black curve), GFP-SBDS-FL in the presence of actinomycinD. We analysed 7–12 cells in 3 independent experiments.

In the presence of actinomycin D, a transcription inhibitor known to affect ribosome production, we observed both an increase in GFP-SBDS-FL nuclear import as well as nuclear export in these cells (Fig. 5C/D/G/H). This suggests that SBDS-FL under steady-state conditions is retained in the nucleus in transcriptional or ribosomal protein complexes, which are disrupted upon actinomycin D treatment. Importantly, these data also provide indirect evidence that GFP-SBDS-FL protein is functional, since interference with ribosome and RNA processing affects mobility of the protein.

In contrast to actinomycin D, treatment of cells with the translation inhibitor cycloheximide did not affect nuclear export of SBDS. However, an increase in GFP-SBDS-FL nuclear import rate was observed in the presence of cycloheximide, suggesting that GFP-SBDS-FL in the cytoplasm is indeed retained in translation-related protein complexes (Fig. 5B/D/F/G). Thus, these data underline the physiological evidence of presented data.

## Discussion

### SBDS protein domains dictate subcellular localization and mobility

**Figure 6:**
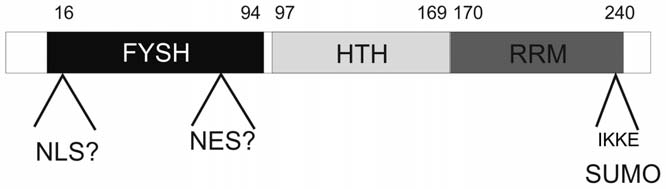
SBDS protein model. Model of the SBDS protein based on our live cell imaging and localization data. Our data suggest that the SBDS N-terminus contains nuclear localization motifs, that the FYSH domain contains a potential Nuclear Export Sequence (NES). Furthermore, we have identified a SUMO consensus sequence (ΨKxE) within the SBDS C-terminal RNA recognition motif (RRM) that is involved in regulating SBDS intracellular mobility.

Our data has provided interesting insights into the SBDS protein domains that dictate subcellular localization and mobility. As indicated schematically in the SBDS protein model (Fig. 6), the SBDS protein contains three domains: an N-terminal FYSH domain, a central helix-turn-helix domain and a C-terminal RNA-recognition motif (RRM) [10], [11]. Our studies have identified three distinct regions within these domains that seem to play a role in SBDS intracellular localization and/or mobility.

First, in the SBDS N-terminal FYSH domain we observed nuclear localization activity. Our localization and mobility data revealed that the GFP-SBDS-K62 and GFP-SBDS-C84 were localized and retained in the nucleus to a greater extent than free GFP (Table 1), suggesting that the SBDS N-terminus contains a potential nuclear localization signal (NLS).

Next, we observed nuclear export activity in the FYSH domain within the region of amino acids 75–85. In our live cell imaging experiments we observed that export rate of SBDS Δ1–75 and SBDS Δ1–65 was higher than SBDS Δ1–85. Also the nuclear-cytoplasmic ratio of GFP-fluorescence of these GFP-tagged SBDS Δ1–75 and SBDS Δ1–65 was significantly lower as compared to SBDS Δ1–85. Together these data suggest that GFP-SBDS Δ1–65 and Δ1–75 contain a nuclear export signal (NES) that is lacking in the GFP-SBDS Δ1–85. However, analysis of the SBDS amino acid sequence did not reveal a known consensus nuclear export signal (NES) sequence. Possibly, so far unidentified, protein-protein interactions with this SBDS domain could be contributing to enhanced export of GFP-SBDS Δ1–75 and SBDS Δ1–65. Additional studies will be required to identify the exact amino acid sequence containing this NES activity and/or the possible protein-protein interactions that may affect cytoplasmic localization of SBDS.

Finally, our data showed that the SBDS C-terminus is playing a dominant role in cytoplasmic localization, since deletion of 32 amino acids of the SBDS C-terminus (i.e. SBDS-R218) result in a prominent nuclear localization. More detailed analysis revealed that the amino acids R218 to L234 in the SBDS protein were important for subcellular localization. The SBDS-L234 mutant protein that lacks the 16 most C-terminal amino acid residues has a similar subcellular distribution as SBDS-FL, but displays higher intracellular mobility as compared to SBDS-FL. This suggests that SBDS amino acids 234–250 play a role in intracellular trafficking, possibly due to protein-protein interactions or alternatively due to intramolecular interactions.

### SBDS sumoylation regulates intracellular mobility

Post-translational protein modification, including ubiquitination and sumoylation, is an important reversible cellular mechanism to change protein function, activity and/or localization [30]–[35]. Sumoylation of proteins was first reported for RanGAP1 and this was shown to affect nucleo-cytoplasmic shuttling of the protein [35]. Currently, 3 different SUMO proteins have been identified in vertebrates. Progress in finding SUMO substrates has been hampered by the fact that generally only a small portion of the protein pool is sumoylated and that SUMO conjugates are rapidly removed by isopeptidases upon cell lysis [30]–[34].

We identified by *in silico* analysis several consensus sumoylation sites in the SBDS protein of which a ΨKxE consensus sumoylation site is located in the SBDS C-terminus. We showed that a fraction of the SBDS protein pool is sumoylated. Site-directed mutagenesis destroying this C-terminal sumoylation site resulted in a clear change in the SBDS protein mobility. Interestingly, SBDS sumoylation of the mutated SBDS isoforms was decreased, but not completely abolished, suggesting that other SBDS protein regions are also sumoylated. Noteworthy is that the patient-derived SBDS protein variants lack the C-terminal sumoylation target sequence. It can be speculated that the disturbed protein motility regulation is contributing to the deregulated SBDS protein function in SDS-patients.

### Live cell imaging studies suggest a role for SBDS related to ribosomal function

To date, different cellular functions have been proposed for SBDS, among which are: (1) a role in migration, (2) a role in mitotic spindle stability and function, and (3) a role in ribosomal processing and/or transport. Consistent with the proposed role in migration, Wessels et al. have shown that GFP-SBDS is localized at the cellular periphery of the leading edge in migrating *Dictyostelium* [38]. Moreover, SDS leukocytes were reported to have a subtle chemotactic dysfunction [5], [6], [38] as well as F-actin polymerization and cellular polarization defects [39]. More recent reports revealed that SBDS is located at the mitotic spindle [17], [18]. Most prominently are the reports showing that SBDS is biochemically involved in ribosome maturation and/or RNA processing [10]–[15], [36]–[38]. The crystal structure of the *A. fulgidus* SBDS ortholog revealed that SBDS contains three distinct protein domains, including possible RNA or DNA interaction motifs [10], [11]. Consistent with this, Menne et al. have shown that the yeast SBDS ortholog, Sdo1, has a role in ribosome maturation, as was confirmed for human SBDS [12], [15]. Recently, several SBDS binding partners with diverse molecular functions were identified using affinity capture and mass spectrometry. These binding proteins include components of the large ribosomal subunit and proteins involved in DNA repair [40]. Our data indicate that, irrespective the preferred binding partner, under steady state conditions, SBDS seems not to be an efficient shuttling protein transporting RNA or ribosomal elements from the nucleus to the cytoplasm or vice versa.

Another nucleolar ribonucleoprotein, nucleophosmin (NPM), that has been previously proposed to interact with SBDS [15], was shown to shuttle between the nucleus and cytoplasm in heterokaryon cellular experiments [41]. To investigate whether trafficking dynamics of SBDS and NPM, indicative for a protein-protein interaction, would be similar, we performed live cell imaging for GFP-NPM. The localization of GFP-NPM was restricted to the nucleoli and therefore we were unable to perform live cell imaging experiments for nucleo-cytoplasmic shuttling. However, we observed that GFP-NPM can move freely between nucleoli, suggesting that NPM, similar as SBDS, is not incorporated into large rigid protein complexes (data not shown). Altogether, in our experiments, SBDS and NPM have strikingly different intracellular localization, which is not supportive of an extensive interaction between SBDS and NPM. Interactions with the aforementioned binding partners from the SBDS-interactome and following co-immunoprecipitation studies [40] would be of interest in future studies on co-localization with SBDS in live cell-imaging studies.

Interestingly, SBDS nuclear export was enhanced upon blockade of cellular transcription, suggesting that SBDS might be present in a transient manner in nuclear ribosomal protein complexes that prevent SBDS transport to the cytoplasm. Actinomycin D treatment of cells results, besides an inhibition of transcription, also in a loss of the nucleolar structures. Despite the fact that we did not commonly observe enriched nucleolar localization of SBDS, our live cell imaging data suggests that SBDS is associated with nucleolar complexes, which are disrupted upon actinomycin D treatment. Also translational inhibition with cycloheximide affects SBDS nucleo-cytoplasmic transport, again suggesting that SDBS in the cytoplasm is also interacting with ribosomal proteins.

In conclusion, our live cell imaging data are consistent with a cellular role for SBDS in ribosome function as previously proposed by several studies [10]–[15], [36]–[38], and are the first to describe at a molecular biological level the differences in protein characteristics between full-length SBDS and SDS-patient derived SBDS protein isoforms.

## Materials and Methods

### Cell culture and transfections

HeLa cells were cultured in IMDM with 10% FCS, penicillin (200 µg/ml), streptomycin (200 µg/ml) and L-glutamine (4 mM). Cells were transfected with Fugene6 Transfection Reagent (Roche) according to manufacturer's instructions and analysed 24–48 hours after transfection.

To disrupt the cytoskeleton cells were treated with either 500 ng/ml nocodazole (Sigma) or with 1 µg/ml cytochalasin D (Sigma) for 30 minutes prior to live cell imaging. To inhibit transcription or translation, cells were treated with 50 ng/ml Actinomycin D (Sigma) or 10 µg/ml cyclohexamide for 2 hours prior to live cell imaging. Imaging was performed in the presence of these inhibitors.

### Cloning of SBDS transcripts

SBDS was PCR amplified from leukocyte cDNA from healthy volunteers with the following primers: SBDS forw 5′-GAGATCGGATCCTCGATCTTCACCCCCACC-3′ and SBDS rev 5′-GAGATCGTCGACTCATTCAAATTTCTCATCTCCT-3′. SBDS cDAs were cloned into pEGFP-C1 vector (Clontech) or pCDNA3.1 vector containing a hemagglutinin (HA) tag. Sequences of the primers that were used to generate SBDS mutant constructs are listed in Supplementary Table S1. All constructs described in this manuscript were sequence verified.

### Western Blotting and pull-down assays

Cells were lysed with 20 mM Tris-Cl pH 7.4/135 mM NaCl/1.5 mM MgCl2/1%TritonX-100/10%glycerol in the presence of protease inhibitors (Complete EDTA-free (Roche)). Proteins were separated on SDS-PAGE and blotted against PVDF membrane (Biorad). Blots were blocked and incubated overnight with the primary antibody followed by incubation with HRP-labelled secondary antibodies (Amersham) and Enhanced Chemiluminescence detection (Pierce).

To detect sumoylated SBDS, Hela cells were co-transfected as described above with HA-SBDS and 6xHis-tagged Sumo1 or 6xHis-tagged Sumo2. Cells were 24 hours after transfection washed with PBS (containing Mg^2+^ and Ca^2+^) at roomtemperature and lysed for 5 minutes in Urea buffer (20 mM Tris–HCl, pH 7,5 200 mM NaCl,10 mM Imidazol, 0.1% Triton X-100 and 8M urea). Cells were scraped, collected and incubated for 10 min at 37°C and centrifuged 5 min at 14000x rpm (RT), after which the supernatant was incubated with 25 µl of prewashed, blocked (200 µg/ml BSA for 1 hr at roomtemperature) Talon beads (Clontech) at roomtemperature for 1,5 hr while rotating. Beads were washed 5 times with Urea buffer and resuspended in Laemlli sample buffer and boiled for 5 minutes at 95°C. Sumoylated HA-SBDS-FL was detected by anti-HA Western-blot analysis.

Antibodies used were anti-GFP (JL-8;Clontech), anti-beta-actin (AC15; Sigma), anti-HA (Y11, Santa Cruz; 12CA5, hybridoma cells were a kind gift of B.Burgering), anti-SBDS (rabbit polyclonal [18]) and anti-Sumo antibodies (kind gift from A.Vertegaal).

### Immunofluorescence

Cells were grown on glass coverslips and transfected as described above. Cells were fixed 16–24 hours after transfection with 4% paraformaldehyde/PBS and permeabilized with NETGEL (50 mM Tris pH 7.4/150 mM NaCl/5 mM EDTA/0.05% NP-40/0.25% gelatine/0.02% NaN3). Cells were stained with the primary antibody overnight, rinsed with PBS/0.5%Tween, incubated with an Alexa488 or Alexa543 conjugated secondary antibodies (Molecular Probes) and counterstained with propidiumiodide (PI) or TO-PRO-3 iodide (Molecular Probes) to visualize the nucleus. Cells were washed with PBS/0.5% Tween and embedded with Mowiol 4–88 (Calbiochem). Pictures were made with a Zeiss LSM 510 META CLSM microscope with Zeiss 65x oil objective at room temperature and processed with LSM 510 and/or Zen 2007 software. GFP intensity was determined with ImagePro or Zen 2007 software.

### Live cell imaging

HeLa cells were grown on 30 mm round Menzel-Glaser glass coverslips, transfected with indicated GFP-tagged SBDS constructs as described and used for live cell imaging 16–24 hours after transfection. Cells were transferred to a POC-mini imaging chamber (Carl Zeiss) and supplied with phenol red-free DMEM/F12 (1∶1) containing 10% FCS, pencillin and streptomycin. Imaging was performed in a conditioned chamber at 37°C with 5% CO_2_ with a Zeiss confocal microscope (LSM 510 META, Zen 2007 software) and Zeiss 63x oil objective.

General FRAP settings were as follows. The 488 nm laser output for all the experiments was set at 50% with bleach power at 100% and 5 images of 500 msec were acquired prior to bleaching. Imaging was performed at 0.5% laser output and 45–445 images of 500 msec were obtained after bleaching. GFP was measured with an optical slice of 1 µm.

To determine intra-nuclear SBDS dynamics a rectangle area (4 µm width) was bleached for 20 iterations and FRAP measurements were performed for 50 images of 500 msec each. To determine intra-cytoplasmic SBDS dynamics a circular area (max 20% of the total cell volume) was bleached for 50 iterations and FRAP measurements were performed for 50 images of 500 msec each. To determine nuclear import, an oval area that covers most of the nucleus was bleached for 50 iterations and a total of 100–250 images of 500 msec each were taken. With Zen 2007 software the average of the absolute fluorescent intensity is measured for the bleach region, the nucleus, the cytoplasm and the background. Additionally, GFP intensity in a control cell was quantified verify that imaging did not cause additional bleaching.

FLIP laser settings for bleaching and imaging are similar for FRAP, except that a circular bleach region in the cytoplasm covered a maximum of 20% of the total cell volume. The first bleach was made after 5 recorded images and bleaching was repeated after every 10 images of 500 msec each. Bleaching is performed with 25 iterations per bleach and a total of 250 images are acquired.

For statistical analysis, background correction was performed. Each different cellular area was normalized to the initial nuclear fluorescent intensity by using the average of nuclear intensity of the 5 images prior to bleaching. Then the average fluorescent intensity and standard error of the mean (sem) is calculated for each time point. Results are presented as the relative fluorescent nuclear/cytoplasm ratio or cytoplasm bleach/cytoplasm non-bleach ratio.

## Supporting Information

Figure S1 **HA-tagged SBDS proteins localize in a similar manner as GFP-SBDS proteins.** (A) Schematic overview of the HA-tagged SBDS constructs (B) Western blot analysis shows that HA-tagged SBDS proteins have the expected molecular sizes of 32 kDa, 14 kDa, 28 kDa and 22 kDa for the HA-SBDS-FL, HA-SBDS-C84, HA-SBDS-R218 and HA-SBDS Δ1-85 respectively. Upper panel shows anti-HA staining and lower panel actin staining as a loading control (C) Representative pictures of the intracellular localization of the HA-tagged SBDS proteins. Upper panel shows propidiumiodide nuclear staining and bottom panel shows HA-SBDS staining. White bar represents 10 µm. (D) Average ratio of the nuclear/cytoplasmic HA fluorescence intensity for the different HA-tagged constructs. (FL n = 30 cells, C84 n = 35 cells, R218 n = 33 and Δ1–85 n = 12 cells in 2 independent experiments). HA-SBDS-K62 was excluded from this analysis due to the low expression levels.
(TIF)

Figure S2 **GFP-SBDS-FL mobility characteristics.** (A) Average of the relative fluorescence intensity of GFP-SBDS-FL in the nucleus (grey line) and the cytoplasm (black line) during 5 minutes after photobleaching of the nucleus. After photobleaching the amount of GFP-SBDS-FL in the nucleus and cytoplasm does not change, showing that there is no nuclear import of GFP-SBDS-FL. (n = 13 cells in 2 independent experiments; error bar indicates s.e.m.). (B) Average of the relative fluorescence intensity of GFP-SBDS-FL in the cytoplasm at the photobleach area (black line; cyto bleach) and in another non-bleached cytoplasmic reveals that GFP-SBDS-FL fluorescence intensity increases rapidly in the photobleach area at the expense of GFP-SBDS-FL fluorescence in other parts of the cytoplasm. Hence, GFP-SBDS-FL is mobile in the cytoplasm (n = 14 cells in 3 independent experiments).
(TIF)

Figure S3 **SBDS C-terminus affects intracellular transport.** (A) FRAP analysis showing the average nuclear/cytoplasmic GFP ratio for GFP-SBDS-FL, GFP-SBDS-K225-226R and GFP-SBDS-E227Q. We analysed 6-13 cells per construct in 2–4 independent experiments. (B) FLIP analysis showing the average nuclear GFP intensity for GFP-SBDS-FL, GFP-SBDS-K225-226R and GFP-SBDS-E227Q. 7–11 cells per construct in 2–3 independent experiments were analysed. (C) Average ratio of the nuclear/cytoplasmic GFP fluorescence intensity for the different GFP-tagged constructs. Error bar indicates s.e.m. (FL n = 21 cells, K225-226R n = 41 cells, E227Q n = 24 cells in 2–3 independent experiments).
(TIF)

Table S1 **Primer sequences.**
(DOCX)
