# Supplementary material for: Altered Intracellular Localization and Mobility of SBDS Protein upon Mutation in Shwachman-Diamond Syndrome
Source: PLoS One. 2011 Jun 13;6(6):e20727. doi: 10.1371/journal.pone.0020727 (PMC3113850; doi:10.1371/journal.pone.0020727)
Supplement: Table S1 — Primer sequences. (DOCX) [file pone.0020727.s004.docx]

**Supplementary Table 1.** Primer sequences used for cloning of the various SBDS constructs used in this study.

| primer name | construct name | primer sequence |
| --- | --- | --- |
| SBDS-FL forw | FL | 5’-gagatcggatcctcgatcttcacccccacc-3’ |
| SBDS-FL rev | FL | 5’-gagatcgtcgactcattcaaatttctcatctcct-3’ |
| SBDS-K62 forw | K62 | 5’-gagatcggatcctcgatcttcacccccacc-3’ |
| SBDS-K62 rev | K62 | 5’-gagatcgtcgacttaggaaacatttacaaacac-3’ |
| SBDS-C84 forw | C84 | 5’-gagatcggatcctcgatcttcacccccacc-3’ |
| SBDS-C84 rev | C84 | 5’-gagatcgtcgacctaacagatttcagtttggtc-3’ |
| SBDS-I86 forw | Δ1-85 | 5’-gagatcggatcccagattttgactaaaggag-3’ |
| SBDS-I86 rev | Δ1-85 | 5’-gagatcgtcgactcattcaaatttctcatctcct-3’ |
| SBDS-R218 forw | R218 | 5’-gagatcggatcctcgatcttcacccccacc-3’ |
| SBDS-R218 rev | R218 | 5’-gagatcgtcgactcagaagcagcccgggtcaatc-3’ |
| SBDS-1-75 forw | A74 | 5’-gagatcggatcctcgatcttcacccccacc-3’ |
| SBDS-1-75 rev | A74 | 5’-gagatcgtcgacttacgcactgatgagatcttcc-3’ |
| SBDS-1-235 forw | L234 | 5’-gagatcggatcctcgatcttcacccccacc-3’ |
| SBDS-1-235 rev | L234 | 5’-gagatcgtcgacttacaaagaacctttgcctttagtt-3’ |
| SBDS-63-250 forw | Δ1-62 | 5’-gagatcggatccaaaggtcaggttgccaaaaag |
| SBDS-63-250 rev | Δ1-62 | 5’-gagatcgtcgactcattcaaatttctcatctcct-3’ |
| SBDS-76-250 forw | Δ1-75 | 5’-gagatcggatcctttggaacagatgaccaaactg-3’ |
| SBDS-76-250 rev | Δ1-75 | 5’-gagatcgtcgactcattcaaatttctcatctcct-3’ |
